# Supplementary material for: Cryptic genetic variation in a heat shock protein modifies the outcome of a mutation affecting epidermal stem cell development in C. elegans
Source: Nat Commun. 2021 May 31;12:3263. doi: 10.1038/s41467-021-23567-1 (PMC8166903; doi:10.1038/s41467-021-23567-1)
Supplement: Supplementary file 1 — Supplementary Information [file 41467_2021_23567_MOESM1_ESM.pdf]

Cryptic genetic variation in a heat shock protein modifies the outcome of a mutation affecting epidermal stem cell development in *C. elegans*

Sneha L. Koneru, Mark Hintze§, Dimitris Katsanos§ and Michalis Barkoulas★

Department of Life Sciences, Imperial College, London SW7 2AZ, United Kingdom.

§ These authors contributed equally to this work.

★Corresponding Author: m.barkoulas@imperial.ac.uk

Supplementary Figures 1-6

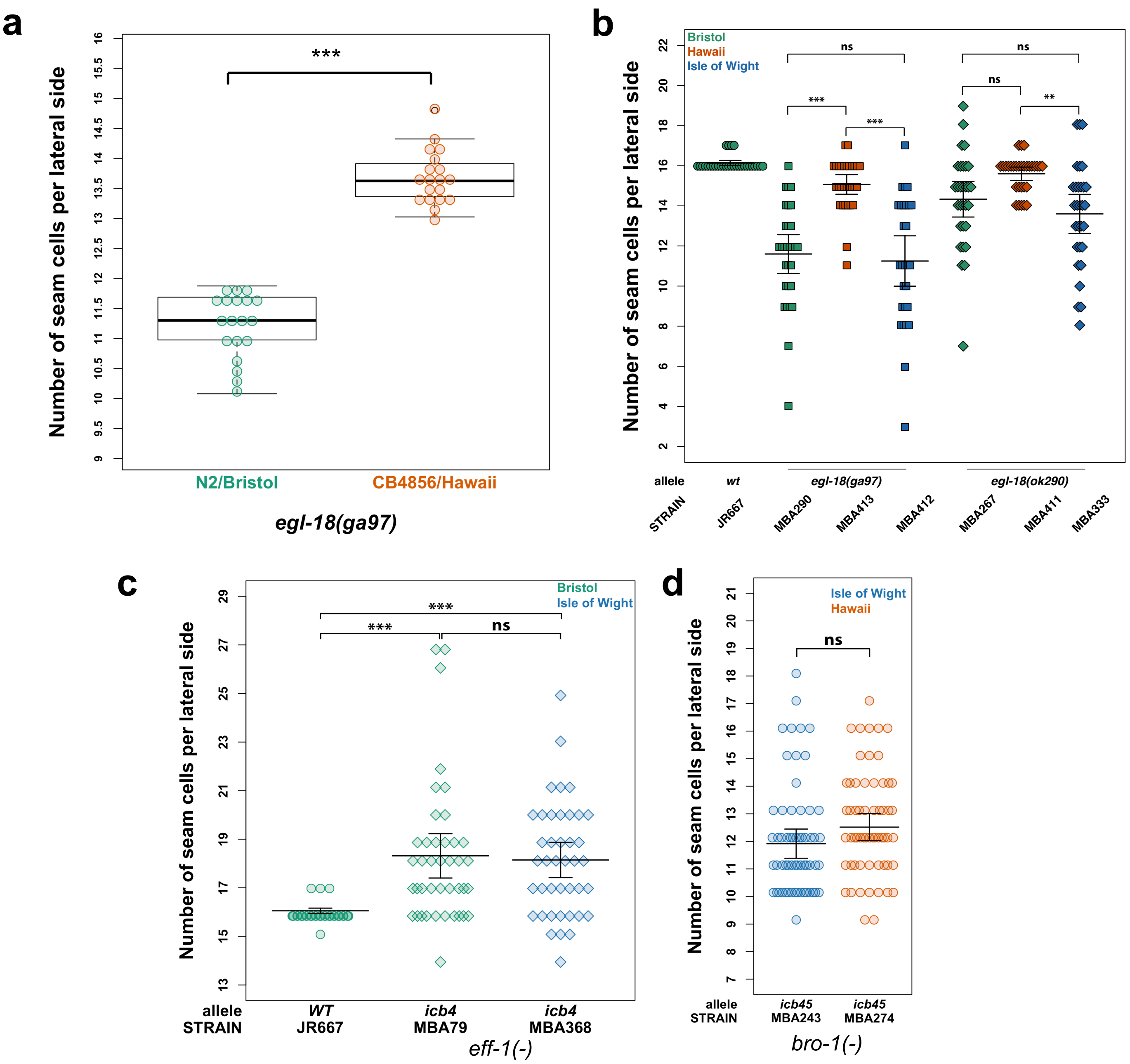
a
Number of seam cells per lateral side
***
N2/Bristol
CB4856/Hawaii
egl-18(ga97)
b
Bristol
Hawaii
Isle of Wight
ns
***
**
allele
wt
egl-18(ga97)
egl-18(ok290)
STRAIN
JR667
MBA290
MBA413
MBA412
MBA267
MBA411
MBA333
c
Bristol
Isle of Wight
***
ns
allele
STRAIN
WT
JR667
icb4
MBA79
icb4
MBA368
eff-1(-)
d
Isle of Wight
Hawaii
ns
allele
STRAIN
icb45
MBA243
icb45
MBA274
bro-1(-)

Figure S1

Supplementary Figure 1: Investigating differences in phenotype expressivity in the seam between N2 and CB4856.

(a) Reproducibility of the difference in average seam cell number between *egl-18(ga97)* mutants in the N2 and CB4856 background. Graph shows the distribution of the average seam cell number in 19 independent experiments (40 animals in each experiment). There is a significant difference using one-way ANOVA $F (1, 36) = 220.15$, $p < 0.0001$). Box plots indicate median (middle line), 25th, 75th percentile (box) and 10th and 90th percentile (whiskers) as well as outliers. (b) The difference in phenotype expressivity is less pronounced when using the weaker allele *egl-18(ok290)*. There was no statistically significant difference in SCN between CB4856 and N2 ($p = 0.06$), but there was a significant difference in SCN between CB4856 and JU2007 carrying the *ok290* mutation ($p = 0.0012$), n = 28 animals for MBA412 and n = 30 animals for all other strains. Seam cell number variance decrease in the CB4856 background for both alleles may be due to the increase in average seam cell number towards the wild-type mean. Statistical comparisons were made with one-way ANOVA followed by Tukey HSD test (c-d) No difference was found in the expressivity of *bro-1* or *eff-1* mutations between wild isolates with one-way ANOVA. There is no statistically significant difference ($p = 0.93$) in SCN between the strains MBA79 (N2) and MBA368 (CB4856) carrying the same putative null allele *icb4* in *eff-1,* n = 37 animals for JR667 and n = 41 animals for MBA79 and MBA368. Similarly, there is no statistically significant difference ($p = 0.09$) in SCN between the strains MBA274 (CB4856) and MBA243 (JU2007) carrying the same deletion allele *icb45* of *bro-1*, n = 60 animals per strain. Error bars in b, c, d show 95% confidence intervals for the mean. Source data are provided as a Source Data file.

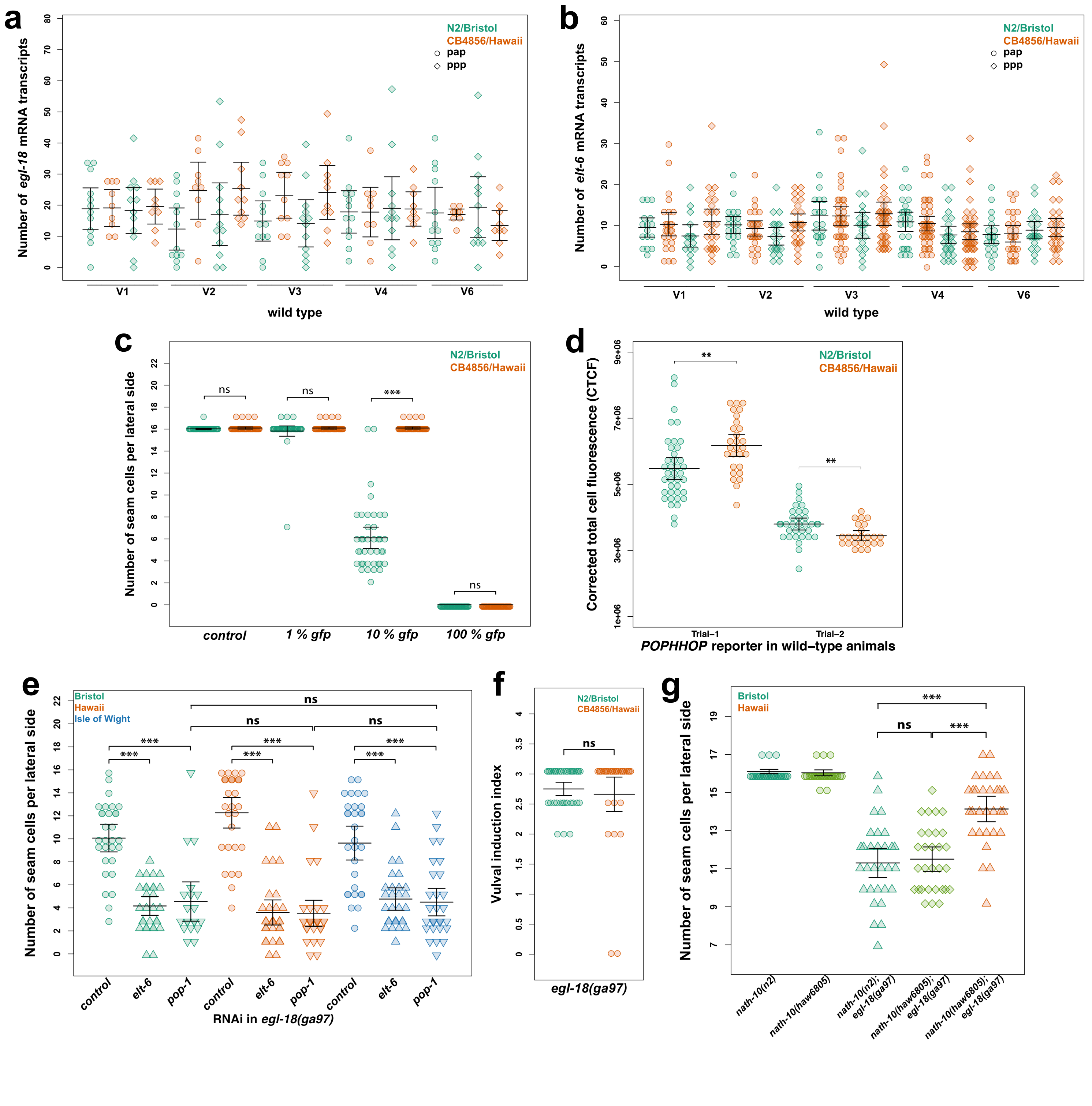
a
Number of egl-18 mRNA transcripts
N2/Bristol
CB4856/Hawaii
pap
ppp
V1
V2
V3
V4
V6
wild type
b
Number of elt-6 mRNA transcripts
N2/Bristol
CB4856/Hawaii
pap
ppp
V1
V2
V3
V4
V6
wild type
c
Number of seam cells per lateral side
N2/Bristol
CB4856/Hawaii
ns
ns
***
ns
control
1 % gfp
10 % gfp
100 % gfp
d
Corrected total cell fluorescence (CTCF)
N2/Bristol
CB4856/Hawaii
**
**
Trial−1
Trial−2
POPHHOP reporter in wild−type animals
e
Number of seam cells per lateral side
Bristol
Hawaii
Isle of Wight
ns
ns
ns
***
***
***
***
***
***
control
elt-6
pop-1
control
elt-6
pop-1
control
elt-6
pop-1
RNAi in egl-18(ga97)
f
Vulval induction index
N2/Bristol
CB4856/Hawaii
ns
egl-18(ga97)
g
Number of seam cells per lateral side
Bristol
Hawaii
***
ns
***
nath-10(n2)
nath-10(haw6805)
nath-10(n2); egl-18(ga97)
nath-10(haw6805); egl-18(ga97)
nath-10(haw6805); egl-18(ga97)

Figure S2

Supplementary Figure 2: Characterisation of the difference in the outcome of the *egl-18(ga97)* mutation between N2 and CB4856

(a-b) Quantification of *egl-18* and *elt-6* expression in wild-type N2 and CB4856 at the late L2 stage (n=120 and 203 cells in N2; n=93 and 320 cells in CB4856 for *egl-18* and *elt-6* quantification respectively). None of the differences between N2 and CB4856 posterior cells are significant with one-way ANOVA followed by post hoc Tukey HSD test. (c) GFP RNAi is used to assess the efficacy of seam cell RNAi in CB4856 and N2 based on *scm::GFP* expression. A significant difference is revealed with one-way ANOVA in 10% dilutions of the GFP dsRNA bacteria with control bacteria (HT115), but not in 1% dilution of GFP bacteria or when applied non-diluted (100% GFP), n = 40 independent animals per strain. (d) Quantification of corrected total fluorescence (CTCF) in N2 and CB4856 wild-type animals carrying the *POPHHOP* reporter. No consistent difference was found among different experimental trials, $n \geq 24$ independent animals per strain, significance is assessed with a Welch two sample t-test. (e) Knockdown of *elt-6* or *pop-1* by RNAi in *egl-18(ga97)* animals masks the differential phenotype expressivity observed. One-way ANOVA showed that SCN was not affected by strain upon *elt-6* RNAi ($F(2, 87) = 1.52$, $p = 0.22$), n = 30 independent animals per strain or *pop-1* RNAi ($F(2, 77) = 0.86$, $p = 0.43$), $n \geq 20$ independent animals per strain (f) No significant difference was found in the vulval induction index between N2 and CB4856 carrying the *egl-18(ga97)* mutation, ($n \geq 31$ independent animals per strain, Welch two sample t-test). (g) The difference in phenotype expressivity is not dependent on a previously known polymorphism in *nath-10*. There is a significant effect of strain on SCN (one-way ANOVA $F(4, 145) = 77.19$, $p < 2.2 \times 10^{-16}$). Post hoc Tukey HSD shows that there is a significant difference between *nath-10(haw6805); egl-18(ga97)* between N2 and CB4856 ($p = 0$). There was no difference

between *nath-10(haw6805); egl-18(ga97)* and *nath-10(N2); egl-18(ga97)* (p = 0.98). n = 30 per strain. In all panels, error bars indicate 95 % confidence intervals around the mean and *** represent p value < 0.0001, ** p < 0.001 or * p < 0.05 by post hoc Tukey HSD test. Source data are provided as a Source Data file.

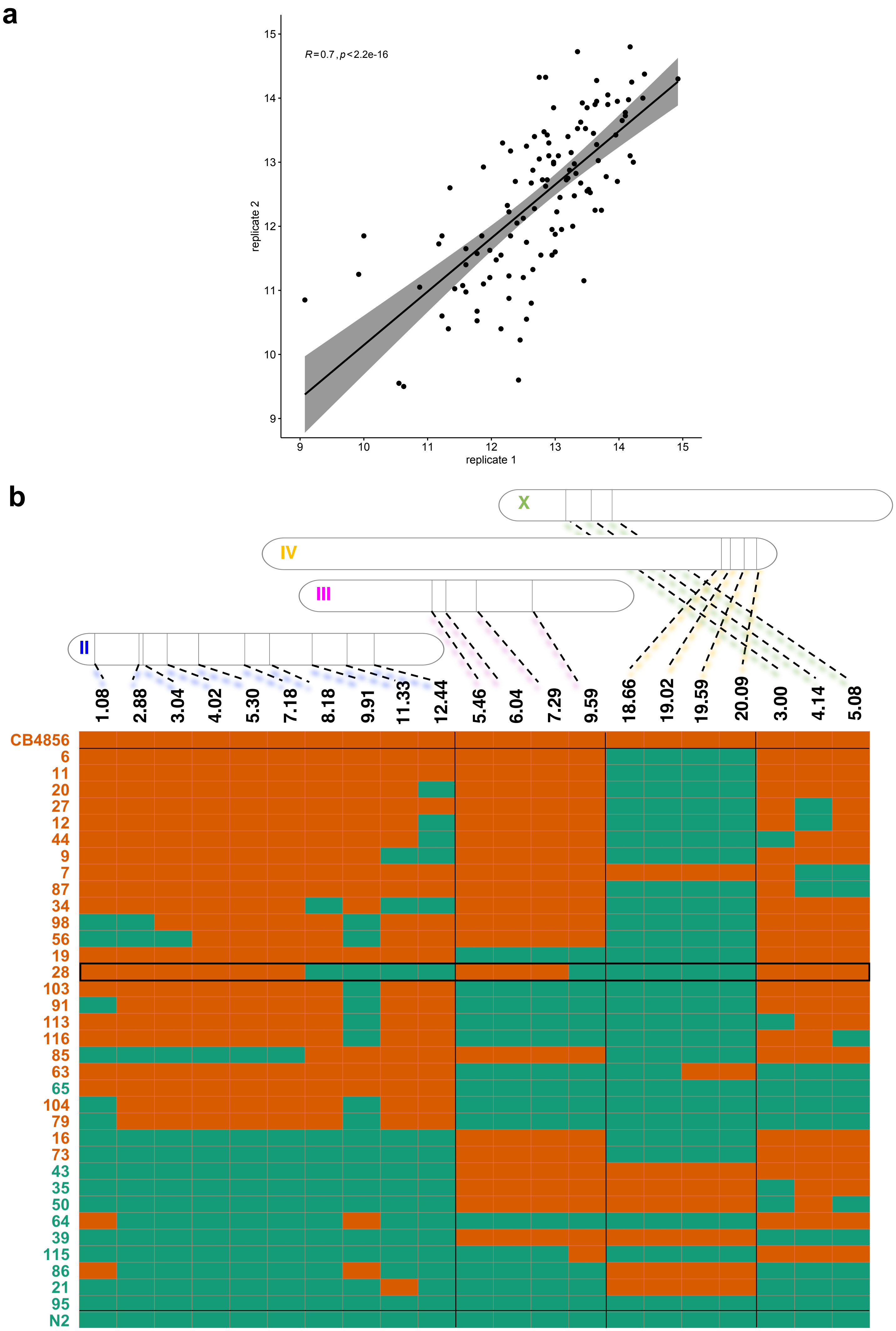

a
R=0.7, p<2.2e-16
replicate 2
replicate 1
9
10
11
12
13
14
15
b
X
IV
III
II
1.08
2.88
3.04
4.02
5.30
7.18
8.18
9.91
11.33
12.44
5.46
6.04
7.29
9.59
18.66
19.02
19.59
20.09
3.00
4.14
5.08
CB4856
6
11
20
27
12
44
9
7
87
34
98
56
19
28
103
91
113
116
85
63
65
104
79
16
73
43
35
50
64
39
115
86
21
95
N2

Figure S3

Supplementary Figure 3: Phenotypic and genetic characterisation of the recombinant inbred lines.

a) High correlation is observed between two independent scorings of RILs (Pearson's correlation coefficient 0.7, $p<2.2 \times 10^{-16}$). Source data are provided as a Source Data file. b) Genotyping of the individual RILs included in the low- and high-bulks using genetic markers**.** Each row represents an individual RIL line and its column a genetic marker on four different chromosomes to infer genetic composition per marker and per RIL. The location of the genetic markers on the chromosomes is shown above the graph. Markers on chromosome II, III, V and X are shown in blue, pink, yellow and green, respectively. Green and orange tiles represent N2 and CB4856 genomes respectively. RILs are ranked in descending order based on number of CB4856 genomic fragments they carry. RIL numbers are colour-coded based on whether they were included in the low-bulk (N2-like, green) or high-bulk (CB4856-like, orange). Note that 24/24 and 10/22 lines were used in this experiment. RIL-28, which was used for producing near isogenic lines containing QTLs on chromosomes II, III and X is highlighted in the middle.

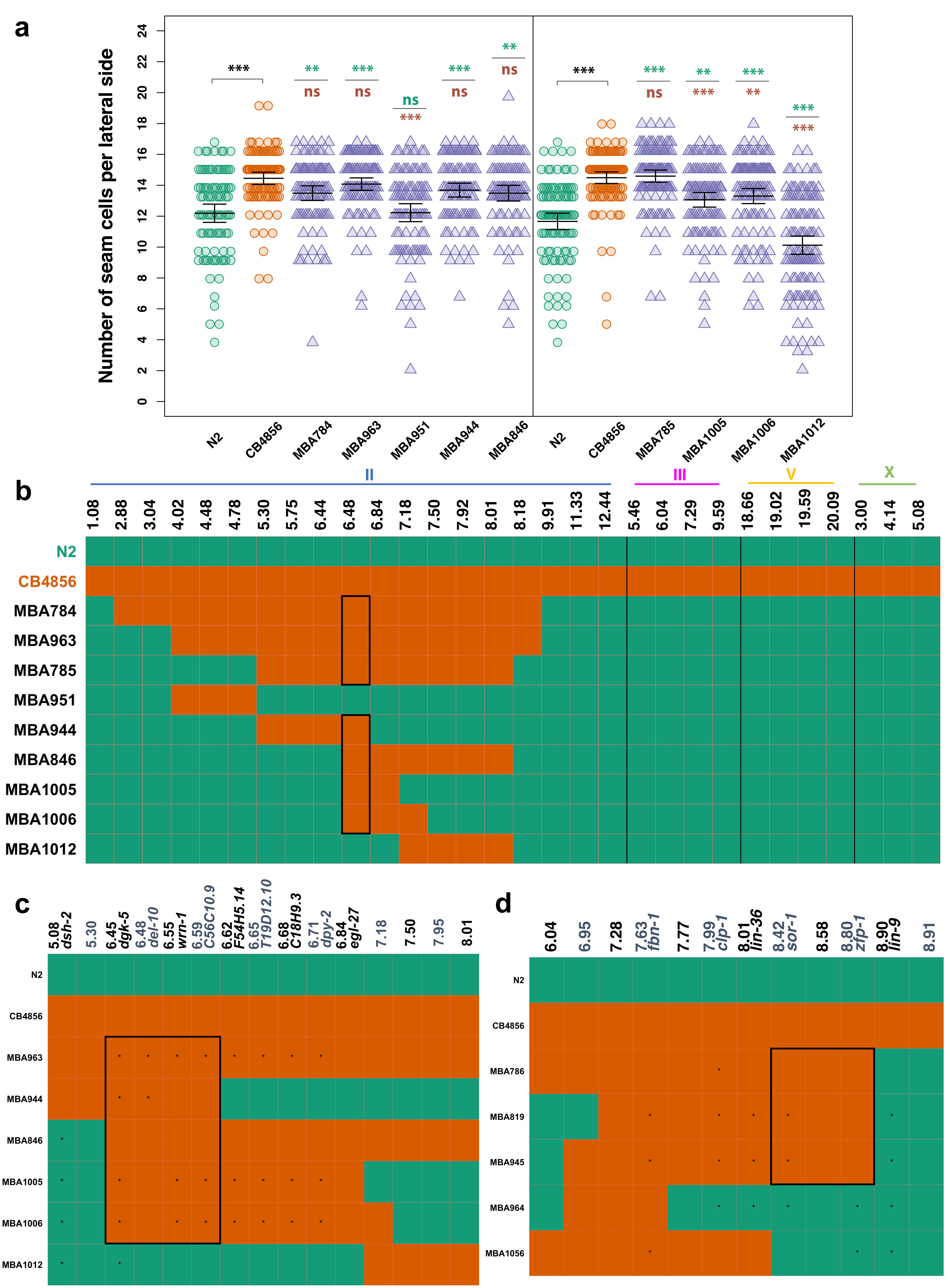

a
Number of seam cells per lateral side
N2
CB4856
MBA784
MBA963
MBA951
MBA944
MBA846
N2
CB4856
MBA785
MBA1005
MBA1006
MBA1012
b
II
III
V
X
1.08 2.88 3.04 4.02 4.48 4.78 5.30 5.75 6.44 6.48 6.84 7.18 7.50 7.92 8.01 8.18 9.91 11.33 12.44 5.46 6.04 7.29 9.59 18.66 19.02 19.59 20.09 3.00 4.14 5.08
N2
CB4856
MBA784
MBA963
MBA785
MBA951
MBA944
MBA846
MBA1005
MBA1006
MBA1012
c
5.08 dsh-2
5.30
6.45 dgk-5
6.48 del-10
6.55 wrn-1
6.59 C56C10.9
6.62 F54H5.14
6.65 T19D12.10
6.68 C18H9.3
6.71 dpy-2
6.84 egl-27
7.18
7.50
7.95
8.01
N2
CB4856
MBA963
MBA944
MBA846
MBA1005
MBA1006
MBA1012
d
6.04
6.95
7.28
7.63 fbn-1
7.77
7.99 clp-1
8.01 lin-36
8.42 sor-1
8.58
8.80 zfp-1
8.90 lin-9
8.91
N2
CB4856
MBA786
MBA819
MBA945
MBA964
MBA1056

Figure S4

Supplementary Figure 4: Genotype-to-phenotype analysis of NILs carrying genomic fragments of chromosome II from CB4856.

(a) Seam cell number in near isogenic lines containing various fragments of chromosome II from CB4856 in the *egl-18(ga97)* N2 background. Vertical black line in the graph distinguishes between two independent experiments. In both experiments, one-way ANOVA showed that SCN was significantly affected by the strain (F (6, 635) = 12.25, p < 4.5 x 10^{-13}; F (5, 622) = 51.18, *p* < 2.2 x 10^{-16}, n ≥ 90 animals per strain. Error bars indicate average 95% confidence intervals around the mean and *** *p* < 0.001 or ** *p* < 0.01 correspond to significant differences by post hoc Tukey HSD compared to N2 (green stars) and CB4856 (orange stars) respectively. Source data are provided as a Source Data file. (b) Genotyping of NILs carrying various genomic fragments of chromosome II from CB4856. Each row represents a RIL line and its column its genetic composition at specific genetic markers, the location of which is shown above the graph. Green and orange tiles represent N2 and CB4856 genomes, respectively. Black box in the middle highlights the genomic region that is common to the NILs that convert N2-like to CB4856-like *egl-18(ga97)* phenotype and absent in the NILs that do not. (c) Close-up on chromosome II showing the region that is common between the NILs that convert N2 *egl-18(ga97)* mutant expressivity to CB4856 levels. (d) Close-up of the region on chromosome III, which includes *sor-1* and *hsp-110* is shown for comparison. In both cases, stars indicate inferred genotype for that marker.

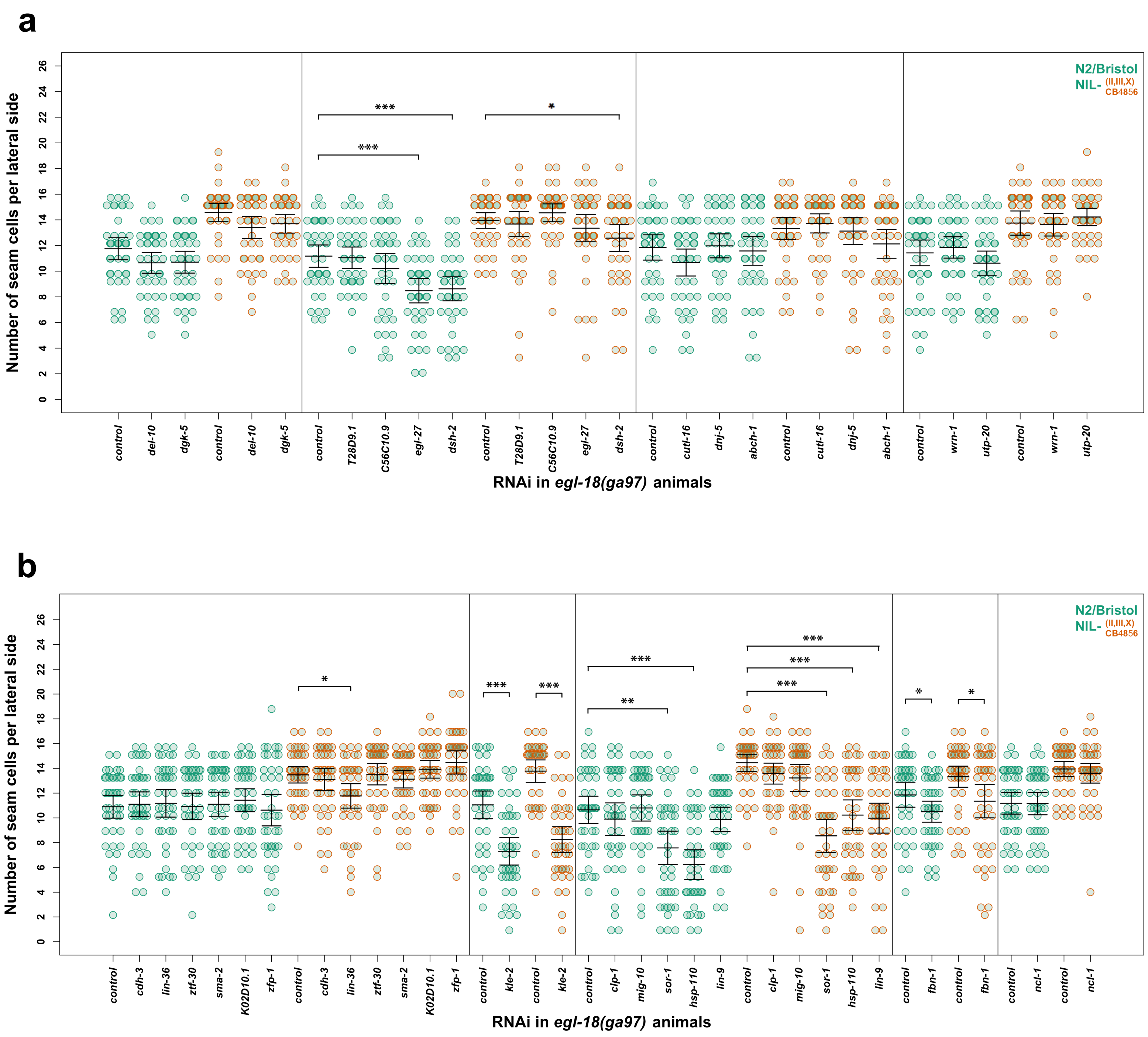
a
Number of seam cells per lateral side
N2/Bristol
NIL- (II,III,X) CB4856
control
del-10
dgk-5
control
del-10
dgk-5
control
T28D9.1
C56C10.9
egl-27
dsh-2
control
T28D9.1
C56C10.9
egl-27
dsh-2
control
cutl-16
dnj-5
abch-1
control
cutl-16
dnj-5
abch-1
control
wrn-1
utp-20
control
wrn-1
utp-20
RNAi in egl-18(ga97) animals
b
Number of seam cells per lateral side
N2/Bristol
NIL- (II,III,X) CB4856
control
cdh-3
lin-36
ztf-30
sma-2
K02D10.1
zfp-1
control
cdh-3
lin-36
ztf-30
sma-2
K02D10.1
zfp-1
control
kle-2
control
kle-2
control
clp-1
mig-10
sor-1
hsp-110
lin-9
control
clp-1
mig-10
sor-1
hsp-110
lin-9
control
fbn-1
control
fbn-1
control
ncl-1
control
ncl-1
RNAi in egl-18(ga97) animals

Figure S5

Supplementary Figure 5: RNAi screen to identify candidate genes in the extended QTL regions of chromosomes II and III.

(a-b) Seam cell number in mutant N2 and MBA848 (NIL carrying the QTLs on chromosome II, III and X) upon RNAi knockdown of candidate genes. (a) Knockdown of most genes at the QTL region on chromosome II did not have a significant effect on the SCN in mutant in N2 and MBA848, except for two genes (*egl-27* and *dsh-2*), which both had a significant effect. (b) Knockdown of two genes at the QTL region on chromosome III (*sor-1* and *hsp-110*) had a significant effect on the SCN in N2 and MBA848 ($F (5, 234) = 10.48$, $p = 8.37 \times 10^{-9}$; $F (5, 234) = 19.65$, $p = 2.47 \times 10^{-16}$, respectively). SCN decreased upon knockdown of *sor-1* and *hsp-110* in both N2 and MBA848 ($p < 0.001$). Knockdown of *lin-9* and *lin-36* showed a significant decrease in SCN only in MBA848. There was a decrease in SCN upon knockdown of *fbn-1* and *kle-2* in both N2 and MBA848. However, these genes were outside the final QTL interval. In a and b, error bars indicate standard deviation around the mean, n = 40 independent animals per strain and *** $p < 0.001$, ** $p < 0.01$, * $p < 0.05$ correspond to significant differences by one-way ANOVA and post hoc Dunnett's multiple comparison test against control RNAi. In both a and b, lines mark independent experiments therefore control treatments are repeated. Source data are provided as a Source Data file.

a

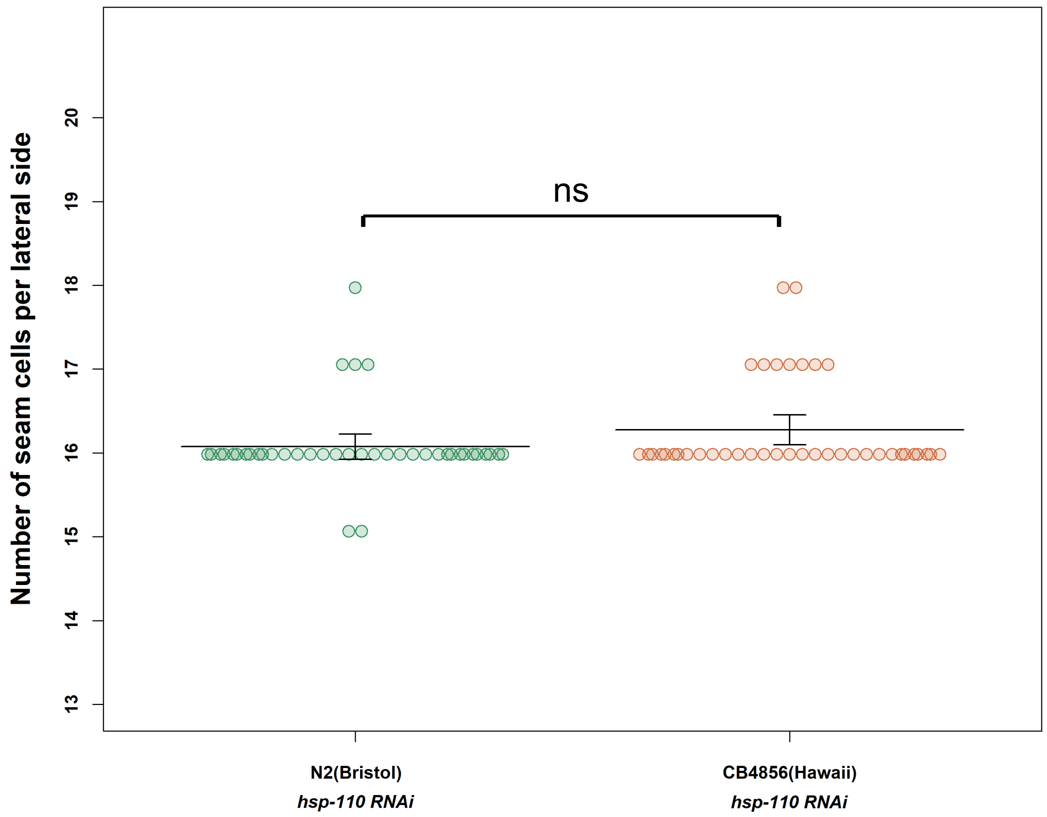
ns
Number of seam cells per lateral side
13
14
15
16
17
18
19
20
N2(Bristol)
hsp-110 RNAi
CB4856(Hawaii)
hsp-110 RNAi

b

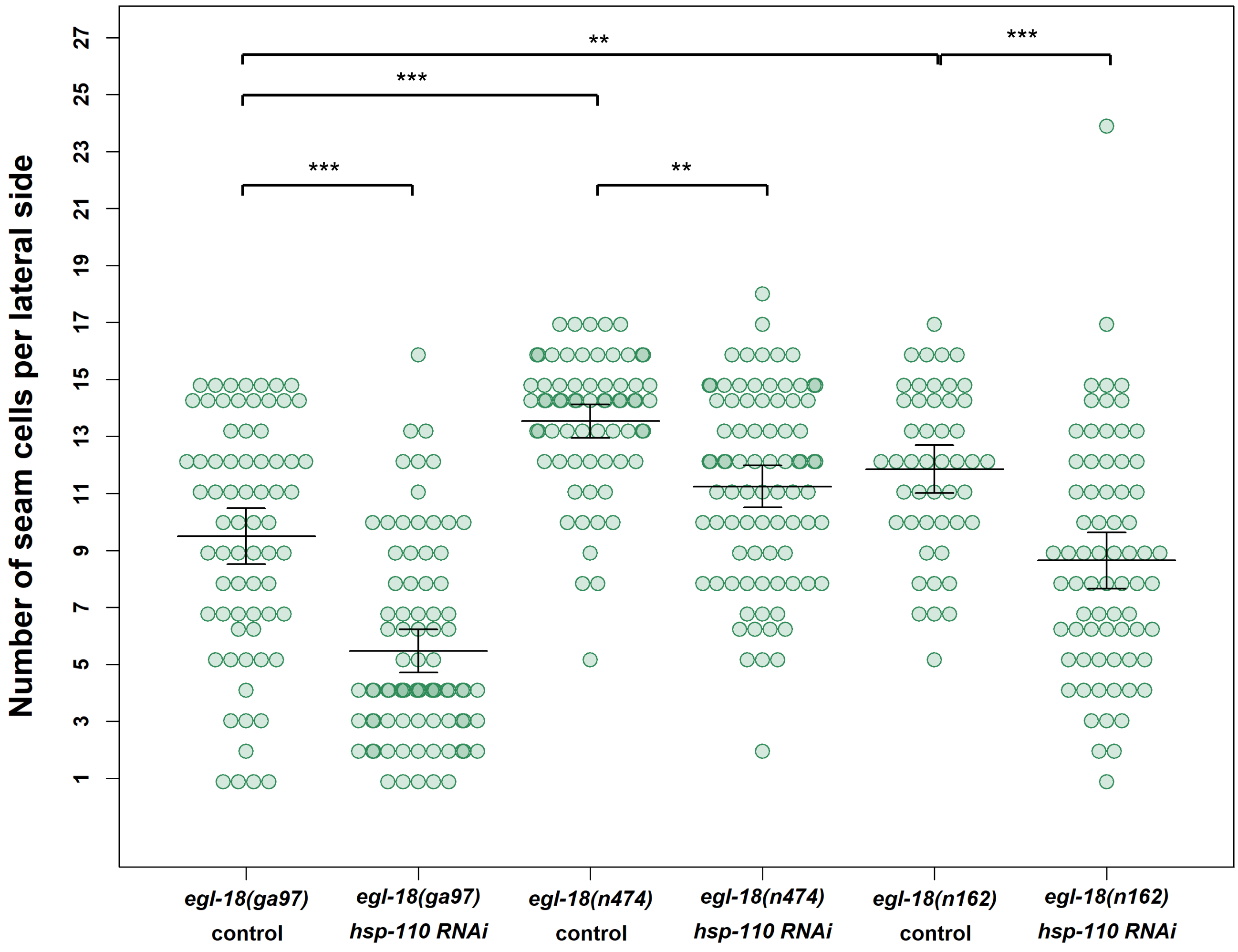
**
***
***
***
**
Number of seam cells per lateral side
1
3
5
7
9
11
13
15
17
19
21
23
25
27
egl-18(ga97)
control
egl-18(ga97)
hsp-110 RNAi
egl-18(n474)
control
egl-18(n474)
hsp-110 RNAi
egl-18(n162)
control
egl-18(n162)
hsp-110 RNAi

Figure S6

Supplementary Figure 6: Interaction between *hsp-110* and other *egl-18* alleles.

a) Down-regulation of *hsp-110* does not change seam cell number in wild-type N2 and CB4856 (n= 40 independent animals); $p = 0.09$ by Welch two-tailed t-test. b) Down-regulation of *hsp-110* decreases seam cell number in other strong loss-of-function alleles of *egl-18* other than *ga97* (n>47 independent animals per strain). Significant differences by one-way ANOVA followed by post hoc Tukey HSD are shown, *** corresponds to $p < 0.001$ and ** to $p < 0.01$. Error bars in a and b indicate 95 % confidence intervals around the mean. Source data are provided as a Source Data file.
