# Supplementary material for: Cryptic genetic variation in a heat shock protein modifies the outcome of a mutation affecting epidermal stem cell development in C. elegans
Source: Nat Commun. 2021 May 31;12:3263. doi: 10.1038/s41467-021-23567-1 (PMC8166903; doi:10.1038/s41467-021-23567-1)
Supplement: Supplementary file 4 — Description of Additional Supplementary Files [file 41467_2021_23567_MOESM4_ESM.pdf]

Description of Additional Supplementary Files

File Name: Supplementary Data 1
Description: Genetic variants within QTL regions on chromosome II and III.

File Name: Supplementary Data 2
Description: Strains used in this study.

File Name: Supplementary Data 3
Description: Primers used in this study (general oligos, genotyping primers and smFISH probes).
